# Digital Therapeutic Care Apps With Decision-Support Interventions for People With Low Back Pain in Germany: Cost-Effectiveness Analysis

**DOI:** 10.2196/35042

**Published:** 2022-02-07

**Authors:** Daniel Lewkowicz, Attila M Wohlbrandt, Erwin Bottinger

**Affiliations:** 1 Digital Health Center Hasso Plattner Institute University of Potsdam Potsdam Germany; 2 Hasso Plattner Institute for Digital Health at Mount Sinai Icahn School of Medicine at Mount Sinai New York City, NY United States

**Keywords:** cost-utility analysis, low back pain, back pain, cost-effectiveness, Markov model, digital therapy, digital health app, mHealth, orthopedic, eHealth, mobile health, digital health, pain management, health apps

## Abstract

**Background:**

Digital therapeutic care apps provide a new effective and scalable approach for people with nonspecific low back pain (LBP). Digital therapeutic care apps are also driven by personalized decision-support interventions that support the user in self-managing LBP, and may induce prolonged behavior change to reduce the frequency and intensity of pain episodes. However, these therapeutic apps are associated with high attrition rates, and the initial prescription cost is higher than that of face-to-face physiotherapy. In Germany, digital therapeutic care apps are now being reimbursed by statutory health insurance; however, price targets and cost-driving factors for the formation of the reimbursement rate remain unexplored.

**Objective:**

The aim of this study was to evaluate the cost-effectiveness of a digital therapeutic care app compared to treatment as usual (TAU) in Germany. We further aimed to explore under which circumstances the reimbursement rate could be modified to consider value-based pricing.

**Methods:**

We developed a state-transition Markov model based on a best-practice analysis of prior LBP-related decision-analytic models, and evaluated the cost utility of a digital therapeutic care app compared to TAU in Germany. Based on a 3-year time horizon, we simulated the incremental cost and quality-adjusted life years (QALYs) for people with nonacute LBP from the societal perspective. In the deterministic sensitivity and scenario analyses, we focused on diverging attrition rates and app cost to assess our model’s robustness and conditions for changing the reimbursement rate. All costs are reported in Euro (€1=US $1.12).

**Results:**

Our base case results indicated that the digital therapeutic care strategy led to an incremental cost of €121.59, but also generated 0.0221 additional QALYs compared to the TAU strategy, with an estimated incremental cost-effectiveness ratio (ICER) of €5486 per QALY. The sensitivity analysis revealed that the reimbursement rate and the capability of digital therapeutic care to prevent reoccurring LBP episodes have a significant impact on the ICER. At the same time, the other parameters remained unaffected and thus supported the robustness of our model. In the scenario analysis, the different model time horizons and attrition rates strongly influenced the economic outcome. Reducing the cost of the app to €99 per 3 months or decreasing the app’s attrition rate resulted in digital therapeutic care being significantly less costly with more generated QALYs, and is thus considered to be the dominant strategy over TAU.

**Conclusions:**

The current reimbursement rate for a digital therapeutic care app in the statutory health insurance can be considered a cost-effective measure compared to TAU. The app’s attrition rate and effect on the patient’s prolonged behavior change essentially influence the settlement of an appropriate reimbursement rate. Future value-based pricing targets should focus on additional outcome parameters besides pain intensity and functional disability by including attrition rates and the app’s long-term effect on quality of life.

## Introduction

### Background

Low back pain (LBP) is the leading cause for worldwide years lost due to disability, and poses a major societal and economic burden with a point prevalence ranging between 9.4% and 37.1% and a lifetime prevalence of up to 85% [1-3]. In Germany, the cost of illness of LBP, referring to International Classification of Diseases-10 code M54 alone, was quantified to be €4.5 billion (~US $5 billion) in 2015 according to the Federal Office of Statistics, which represents 1.3% of the total national health care expenditure [4]. In various cost-of-illness studies, the overall costs of LBP are estimated to be even higher. For example, one study estimated average costs of €1322 (US $1474) per patient per year from a sample of 5650 LBP patients and extrapolated these costs to €48 billion (~US $53.5 billion) for the whole German population [5]. The major cost driver for health care systems lies in the predominantly high portion of indirect costs, which are estimated to range from 55% up to 87% for LBP patients and result from the vast majority of absences from work, leading to productivity losses [6,7]. Conversely, the minor part concerning direct costs quantifies the amount of health care resource utilization such as the number of primary care consultations or prescribed medications. Patients suffering from chronic LBP, which evolves in approximately 10%-15% of all LBP cases, are prone to occasion more than three-times higher costs than those incurred by patients with acute LBP [6,7]. Moreover, an increasing chronic pain grade (eg, as measured with the Von Korff pain scale) has been identified as the strongest predictor of high LPB costs [8]. According to the German Disease Management Guidelines, current treatment recommendations for nonspecific, nonacute LBP include remaining physically active, exercise and educational therapy, and psychosocial interventions. A recent systematic review investigated 15 distinct clinical practice guidelines for the management of nonspecific LBP in primary care from 15 different countries published between 2010 and 2017 [9]. All included guidelines provided recommendations for exercise therapy [9]. Moreover, pharmacological treatment should be reduced to a minimum and should only be prescribed as part of an overall therapeutic concept [9-11].

Digital therapeutic care apps are innovative new treatment programs with a variety of indication-specific video-based exercises and educational material accessible through a smartphone or a web-based app [12]. Recent research endeavors have shown that this multidisciplinary treatment modality can counteract the rising health care expenditure in multiple dimensions [12-14]. First, digital therapy apps provide a scalable and broadly accessible approach, enabling the treatment of LPB in rural areas and when the availability and workload of physiotherapists are limited [15]. Moreover, although stratified care is not yet implemented in routine care in Germany (eg, using the STarT-Back questionnaire), digital therapy apps enable early and immediate utilization for patients at high risk for developing a worsening or chronic condition [16,17]. Digital therapeutic care apps also support self-management and increase the patient’s literacy through in-depth educational information [18]. Hence, these apps can further induce positive reinforcement through personalized decision support that entail motivational automated push notifications or tailored exercise recommendations based on personal preferences [19,20].

By contrast, previous retrospective studies of real-world user-generated data have shown that digital therapeutic care apps may imply low user retention and high early attrition rates [12]. The reasons are not entirely clear, but these drawbacks might mitigate the apps’ overall health and economic benefits [18]. An analytical study conducted by the German Bertelsmann Foundation in 2012 estimated potential cost savings of approximately €3 billion per year based on resolving noncompliance and lack of therapy adherence of chronic LBP patients [21]. Therefore, the economic consequences of reimbursing and prescribing digital therapeutic care apps are unclear, and the tradeoff between positive impact and low engagement rates requires investigation within an economic evaluation. Considering the perpetual rise of health care expenditure in middle-to-high-income countries such as in Germany or the United States reaching 11.9% and 17.7% of the total gross domestic product, respectively, the appropriate allocation of health care resources and the management of cost-effective treatment modalities for LBP need to be analyzed and addressed more profoundly from an economic perspective [22,23]. Furthermore, the new Digital Healthcare Act in Germany allows apps with proven scientific evidence to be part of the reimbursement catalog of the statutory health insurance providers once the app is listed in the Digital Health Applications (DiGa) directory [24,25]. This initiative helps to reduce out-of-pocket costs for patients with LBP by covering digital therapeutic app costs and essentially removing one major barrier for upscaling the utilization of digital health apps. Moreover, a regulatory-driven decision process, including the selection of reimbursable therapy apps, will further increase the transparency for patients concerning which apps already have proven scientific evidence of being effective. Overall, these developments underline the urgency of evaluating the initial pricing of digital therapeutic care apps for people with LBP as well as for the statutory health insurance based on a long-term model-based economic evaluation.

### Objectives

In this cost-effectiveness analysis, we investigated the economic impact of a digital therapeutic care app as an alternative treatment approach to current treatment-as-usual (TAU) practices, including face-to-face (F2F) physiotherapy and concomitant pharmacological treatment. We extrapolated short-term evidence of the impact of a therapeutic care app based on the effectiveness data of a single randomized controlled trial (RCT) [18]. Essentially, we aimed to simulate the divergent tradeoffs between digital therapeutic care and TAU: higher cost per app prescription and higher app attrition rates but also more self-management support to achieve behavior change, versus lower cost for F2F physiotherapy but also fewer possibilities to provide patients with reinforcing education material for coping with LBP. In addition, we simulated the reimbursement of a therapy app based on the current procedure in Germany in a best-practice decision-analytic Markov model and calculated the overall cost utility from a societal perspective. This study provides economic evidence that can inform other researchers and decision-makers, and further addresses the gap in health economic research by performing the first model-based economic evaluation of digital therapeutic care apps for people with nonspecific LBP.

## Methods

### Study Design

We constructed a decision-analytic, discrete-time Markov chain to simulate the long-term effects on treatment and cost outcomes of a digital therapeutic care app compared to TAU for patients with subacute and chronic nonspecific LBP. A Markov chain is a state-transition model that represents a stochastic process in which subsequent events occur with a predefined probability, which is also called the transition probability [26]. In health economic evaluations, these events are defined as health states that represent the patient’s disease process over time. In the simulation, after each model cycle, the patient’s health condition might change, and thus the patient cohort moves around to one or more subsequent health states given a specific transition probability [26].

Our economic evaluation was based on the effectiveness data from the currently only available RCT performed in Germany by Toelle et al [18], in which the impact of a therapy app was investigated without any additional interventions or tools. The authors investigated the effectiveness of an app over 12 weeks and compared it to six F2F physiotherapy sessions combined with online education material. Hence, our model narrows down treatment modalities and compares a therapeutic care app to TAU (ie, F2F individual physiotherapy), both accompanied by general practitioner (GP) and specialist consultations, concomitant pharmacological treatment, and diagnostic procedures. Particularly, we focus on the recurring pain episodes and implications of various treatment attrition rates on health care resource utilization in the primary care setting. However, we chose to exclude inpatient procedures, rehabilitation care, and injection therapy as further treatment modalities for two reasons. The first reason is that none of these interventions is recommended for our target population of LBP patients, and the second is that we lack data to populate the Markov chain and claim that by adding more complex treatment combinations and assumptions, the usefulness of our model would decrease. Moreover, we used quality-adjusted life year (QALY) utility scores to account for health-related quality of life (QoL) effects and express cost in 2021 Euro (€) values (€1=US $1.12). All utility scores and cost data were discounted with a discount factor of 3%. We derived all information regarding the amount of health care resource utilization from other clinical or cost-of-illness studies in the literature. Model simulation and calculations were performed in R using the “heemod: Health Economic Evaluations MODeling” package [27]. Our economic evaluation adheres to all items of the Consolidated Health Economic Evaluation Reporting Standards (CHEERS) statement [28]; also see Multimedia Appendix 1. More detailed explanations and calculations regarding all model input parameters can be found in Multimedia Appendix 2.

### Patient Population

We focused on a specific subcohort of nonspecific LBP patients who are insured via the German statutory health insurance and suffer from subacute or chronic LBP with a greater probability of recurring mild or heavy pain episodes. Particularly, we considered a hypothetical cohort of average 41-year-old participants with a mean BMI value of 24.4 in the intervention group, which was based on the cohort data from our reference RCT study by Toelle et al [18]. According to current clinical guidelines, this patient cohort should be treated with physiotherapeutic-based exercise programs and educational material for ergonomic and health-promoting behavior change, combined with temporary medication therapy for momentary pain reduction [9]. Conversely, we excluded acute LBP patients from this evaluation because, although remaining active is vital in this pain phase, extensive physiotherapy is not recommended for this subgroup [9]. Moreover, we did not intend to evaluate any treatment approach toward patients with existing red flags (eg, osteoporosis or any other severe comorbidities) or with yellow flags (eg, critical psychosocial factors). For instance, depressive disorders are a potential driving cause of the underlying pain condition and an essential predictor for future costs, but this would require additional psychotherapeutic care and involve the consideration of another treatment pathway, which was not the intention of this economic evaluation [6].

### Model Structure and Transition Probabilities

**Figure 1:**
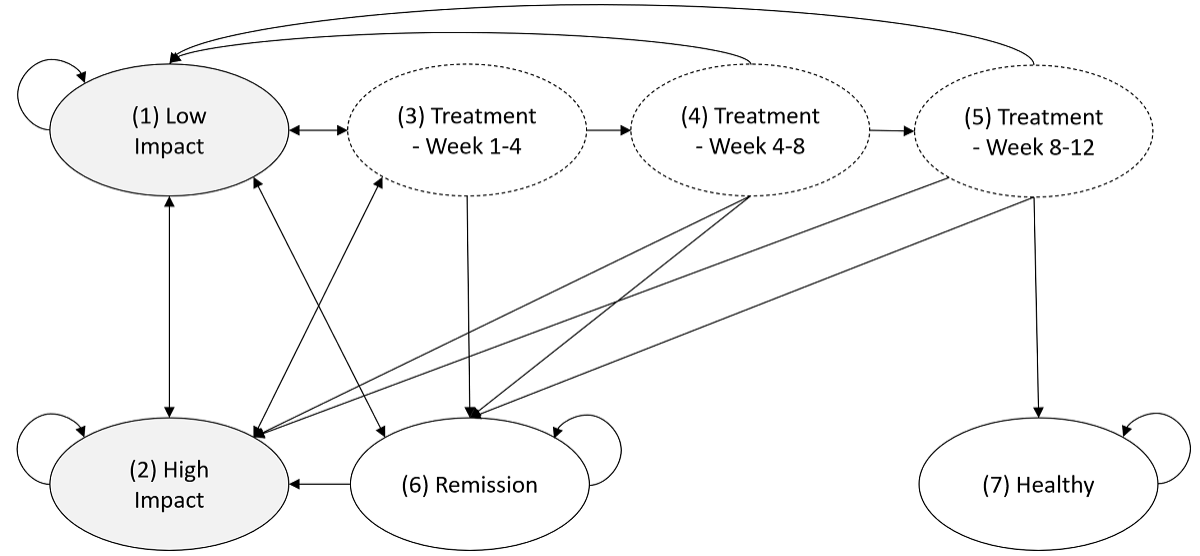
Discrete health state-transition Markov chain with 7 health states.

In our Markov chain model, displayed in Figure 1, we defined a total of seven discrete health states: (1) low impact, (2) high impact, (3) treatment weeks 1-4, (4) treatment weeks 4-8, (5) treatment weeks 8-12, (6) temporary remission, and (7) healthy.

We did not include “dead” as another absorbing state, since LBP is not affiliated with a higher death rate. All-cause mortality does not affect the overall outcome considering our middle-aged population in the defined time horizon of 3 years in the base case. We chose a cycle length of 4 weeks for our simulation for two reasons. First, a 4-week cycle length enables the consideration of three treatment phases, one for each month, which in turn represents the recommended 3-month treatment according to clinical guidelines. Second, recent studies of digital therapeutic care apps as well as the Toelle et al [18] study reported primary and secondary outcome measures based on monthly surveys. Hence, we found data to simulate monthly model cycles, but not enough data to increase the complexity such as by considering a weekly cycle length. We also applied the life-table method to account for corrected state membership counts [29]. Low-impact and high-impact health states (1) and (2) included untreated people with a respectively low or high impact on the QoL resulting from LBP. In the low-impact state, we classified people with low functional impairments and low limiting consequences. By contrast, people classified into the high-impact states were those suffering from high functional disability with moderately to severely limiting LBP. An intermediate impact state was excluded because we have not found sufficient data in the literature to further populate the model toward the relation to the other states. We decided to group the initial starting population based on the severity of symptoms. This approach enabled matching toward health states (1) and (2), which depict the corresponding level of the low and high impact of LBP. We found the Von Korff Graded Chronic Pain Scale to be the best choice for this matching, as this tool enables the classification of the severity of symptoms toward chronological pain grades [3]. For our model, we combined pain grades I and II to correspond to state (1) and the highest two pain grades III and IV to correspond to state (2) by adding up the correlated percentages for each category extracted from a German multiregional survey [3]. Thus, the hypothetical primary care cohort of 10,000 people was allocated to states (1), (2), and (6) with a starting distribution of 5320, 1120, and 3560 participants for each state, respectively [3,5].

Furthermore, we introduced treatment states in the form of three tunnel health states to represent a recommended treatment length of 12 weeks [30]. This approach was found to be highly useful when modeling time-to-treatment variations in a cost-utility analysis for internet-based cognitive behavioral therapy reported by Bauman et al [31]. We thus adopted the methodology of dedicated treatment states to optimize our model regarding the simulation of divergent monthly attrition rates. After each cycle, patients drop out of a treatment program for various reasons (eg, spontaneous remission phase with no present symptoms, stagnation of any health improvements, worsening condition with a fear of movement or pain, or simply a lack of motivation and time) [16,32]. In the RCT [18], for the base case, the attrition rates were 12.5% after both month one and month two in the digital therapeutic care app group, and were 6.5% after month one and 4.3% after month two in the control group. Conversely, in the digital therapeutic care strategy, patients continued treatment with a probability of 87.5% of proceeding to the next cycle, whereas this probability was 93.5% after month one and 95.7% after month two for the TAU strategy.

However, the discontinuation of either treatment strategy does not deduce any insights on the improvement or worsening effect on the patients’ QoL. Therefore, we assumed an equal distribution of the remission rate of 50% among those who quit the program at each time point. According to a long-term cohort study in primary care performed in the United Kingdom, patients with LBP remain in a similar pain trajectory over time without any fluctuating patterns (ie, patients with mild pain will again with high probability experience mild pain in future episodes and vice versa) [33,34]. Following this argumentation, we inferred transition probabilities by proportionately splitting the remaining count of dropouts, meaning that 82.2% of nonremission dropouts will again transfer to the low-impact state group and 17.8% will transfer to the high-impact group [33].

Moreover, nonspecific LBP is characterized as a recurrent disease. Phases of pain and functional disability may frequently occur alternately to a temporal phase of relief [34]. The “remission” state thereby serves as an intermediary simulation approach to include the temporary fluctuating and episodic nature of LBP with different intensities [31,34]. According to a German LBP survey with an adult sample size of 5650, 61.4% of participants experienced pain episodes repeatedly, which we utilized as the transition probability going from “remission” to either a low- or high-impact state [5]. In the case of a recurring episode, we further assumed that the GP or orthopedist will reevaluate the clinical findings through an imaging diagnostic procedure after the patient reenters the treatment pathway [35]. To capture the economic tradeoffs of recurrences and readmissions of patients to primary care, we chose a model time horizon of 3 years.

Finally, we assumed that only patients who underwent treatment and were fully engaged for 3 months can transfer to the “healthy” state [5]. We defined the “healthy” state as not reexperiencing LBP in the scope of this model’s time horizon. Including a health state for both types of remissions, a spontaneous remission in state (6) and a long-lasting pain-free healthy condition in state (7), allowed us to consider the episodic nature of LBP and address those events with distinct transition probabilities, which are drawn from the literature [31]. In the underlying RCT [18], patients received access to the therapy app for 3 months, whereas the control condition only included six physiotherapy sessions. Hence, the 3-month app access duration with proven continuous high user retention affects the maintenance of performing exercises in the long term [18]. Moreover, decision-support interventions in the digital therapeutic care app have a high chance to induce positive behavior change by repeatedly informing and motivating the user through push notifications and in-app on-demand education material [36,37]. We thus derived that in the digital therapeutic care app strategy, the transition probability from the last treatment cycle to “healthy” is 5% higher. All model parameters regarding the transition probabilities and QoL utility scores are summarized in Table 1.

**Table 1:** Model input parameters: transition probabilities and quality of life (QoL) utility scores.

Parameter			Base case^a^			DSA^b^			Reference
		DTC^c^		TAU^d^	Low^e^		High^f^		
**Key transition probabilities**									
	Low to Low	0.16		0.16	—^g^		—	[33,34]	
	Low to High	0.01		0.01	—		—	[33,34]	
	Low to T^h^_W^i^1-4	0.75		0.75	0.60^j^		0.90	Assumption (75%)	
	Low to Remission	0.03		0.03	—		—	[33,34]	
	High to Low	0.02		0.02	—		—	[33,34]	
	High to High	0.08		0.08	—		—	[33,34]	
	High to T_W1-4	0.80		0.80	0.70^j^		0.90	Assumption (80%)	
	T_W1-4 to Low	0.0514		0.0267	—		—	[3,5,12,38]	
	T_W1-4 to High	0.0111		0.0058	—		—	[12,33]	
	T_W1-4 to T_W4-8	0.875		0.935	—		—	[12,18]	
	T_W1-4 to Remission	0.0625		0.0325	0.40^j^		0.60	Assumption (50%)	
	T_W4-8 to Low	0.0514		0.0177	—		—	[12,33]	
	T_W4-8 to High	0.0111		0.0038	—		—	[12,33]	
	T_W4-8 to T_W8-12	0.875		0.957	—		—	[12,18]	
	T_W4-8 to Remission	0.0625		0.0215	0.40^j^		0.60	Assumption (50%)	
	T_W8-12 to Low	0.235		0.235	—		—	[33,34]	
	T_W8-12 to High	0.051		0.051	—		—	[33,34]	
	T_W8-12 to Remission	0.614		0.614	0.583^k^		0.644	[33,34]	
	T_W8-12 to Healthy	0.10		0.05	0.095^k^		0.105	Assumption	
	Remission to Remission	0.386		0.386	0.30^j^		0.46	[5]	
	Remission to Low	0.505		0.505	—		—	[3,5]	
	Remission to High	0.109		0.109	—		—	[3,5]	
**QoL utility scores**									
	Low impact	0.655		0.655	—		—	[18]	
	Higher pain	0.610		0.610	0.5795^k^		0.6405	[39]	
	T_W1-4	0.655		0.655	—		—	[18]	
	T_W4-8	0.699		0.717	—		—	[18]	
	T_W8-12	0.748		0.729	—		—	[18]	
	Remission	0.806		0.806	0.7657^k^		0.8463	[39]	
	Healthy	0.806		0.806	0.7657⁵		0.8463	[39]	

^a^Base case values are listed as the final calculated inputs used in the model. The raw numbers are provided in Multimedia Appendix 2.

^b^DSA: deterministic sensitivity analysis.

^c^DTC: digital therapeutic care.

^d^TAU: treatment as usual.

^e^Low: low-impact low back pain.

^f^High: high-impact low back pain.

^g^not applicable.

^h^T: treatment.

^i^W: week.

^j^Values are shown in raw numbers (ie, before the actual relative transition probability was calculated). All absolute transition probabilities are listed in Multimedia Appendix 2.

^k^Based on a –5% to +5% interval range.

### Measurement of Effectiveness: QoL

We retrieved the effectiveness data concerning the three treatment states from the reference RCT of Toelle et al [18]. In the intervention group, the authors found a significant improvement in the health-related QoL scores in both groups but no significant difference between groups, neither regarding pain levels nor QoL measurement [18]. However, after 12 weeks, patients in the app group had a higher mean QoL score compared with that of patients in the control group. For the QoL measurement, we adapted the QoL outcome results based on the Veterans RAND 12-Item Health Survey (VR-12) [18]. We gratefully retrieved the VR-12 data from the authors and calculated the single-utility index Veterans RAND 6-Item Health Survey (VR-6D) scores according to the approach proposed by Selim et al [40,41]. The detailed calculation steps for the VR-6D are listed in Multimedia Appendix 2. Notably, we applied QoL data for the remaining health states (2), (6), and (7) based on the Short Form 6-Item Health Survey (SF-6D) from another LBP study [39] because QoL data for these health states were not available from the reference study. We consider this methodological choice applicable because both strategies in our simulation do not differ regarding the QoL measure for these three health states. Consequently, the important difference in QoL outcome occurs only in three treatment states, (3), (4), and (5), for which the QoL data rely on the single study estimates. Even though interchanging different QoL metrics is not recommended in most cases, prior research has shown that the VR-6D and SF-6D are comparable indices with similar utility scores [40].

### Health Care Resource Utilization and Costs

For the evaluation of cost outcomes, we considered LBP-related utilization of health care resources and procedures as well as productivity losses that result from absenteeism from work. The direct cost components in our model include outpatient consultations, app subscription cost, F2F physiotherapy sessions, pharmacotherapy, and diagnostic procedures. Considering the patient visits in primary care, we multiplied the number of GP (“Hausarzt”) and specialist or orthopedist (“Facharzt”) consultations with provider-specific charges according to the German medical fee schedule for physicians (“Einheitlicher Bewertungsmaßstab”: EBM 13211 and 18211) [38,42]. Moreover, participants in the control group of Toelle et al’s [18] RCT received weekly, guideline-conforming individual physiotherapy for six sessions, each at least 20 minutes long. We calculated a charge of €21.11 (Position X0501 in the German “Heilmittelkatalog”) for each session and added the obligatory patient copayments of a one-time €10 prescription charge and an additional 10% own share for each physiotherapy session. Moreover, we extracted data on the utilization of pharmacotherapy and diagnostic procedures from the German cost-of-illness study performed alongside an RCT [6] instead of referring to the reference RCT study. We chose this approach because the reference study only reports on the outcome of the medication quantification scale, which is a value for a patient’s medication profile, and does not include data on the monthly frequency and dose of medication intake. Nonetheless, we adopted the finding that there was no significant between-group difference regarding medication intake [18]. We inflated the reported monthly mean cost of medication intake, including nonsteroidal antiinflammatory drugs as the most frequently used drugs, and monthly mean medical image diagnosing cost per patient to 2021 prices assuming an annual expenditure growth rate of 3% [6]. Since we did not consider patient treatment in inpatient care, surgical or rehabilitation costs were excluded. Finally, we utilized the price for the app’s 3-month subscription in Germany from the DiGa repository, in which another, yet similar in functionality, listed therapy app for people with LBP is currently listed and being reimbursed by statutory health insurance at a price level of €239.96 [43].

We estimated the indirect cost regarding the vocational outcome using the human capital approach. We multiplied the average hourly labor cost with productivity losses due to the LBP-related number of days of absences from work, assuming 21 working days per month and an average monthly gross wage of €3092 in 2020 in Germany [44]. Becker et al [6] reported that for one-third of patients, the mean frequency of short-term productivity losses was 8 days, whereas highly frequent utilizers with 5 or more days off work accounted for 98% of total absenteeism. Another German cost-of-illness study performed by Wenig et al [5] reported a mean value of 13.5 days of sick leave over 3 months. We thus assumed absenteeism to be on average the sum of 8 days for all states after each cycle in the model, which is also in alignment with a systematic review that reported a median duration of work absence of 5 to 28 days in the workplace samples [45]. However, we did not include productivity losses resulting from employees remaining at work with restricted operating activity, so-called presenteeism [5]. Furthermore, patients in the remission state do not cause any additional expenditure since we assumed that no follow-up pharmacotherapy nor primary care consultation is utilized in that health condition phase. All expenditure-related data that we used to populate the model as well as the mean monthly cost per patient and cycle are reported in Table 2.

**Table 2:** Model input: health care resource utilization and cost parameters (in Euro: €1=US $1.12).

Parameter		Unit	Base case	Deterministic sensitivity analysis		Reference
				Low	High	
**Direct cost**						
	DTC^a^ app	One-time access (for 3 months)	239.96	99.96^b^	299.96^b^	[43]
	GP^c^	Consultation	20.47	—^d^	—	[5]
	Orthopedic specialist	Consultation	21.36	—	—	[38,42]
	Physiotherapist	Session	21.11	—	—	[38,42]
	Physiotherapist	Cycle (6 session)	149.33	102.88^e^	288.65^e^	[38,42]
	Pharmacotherapy	Per cycle	16.81	—	—	[6]
	Diagnostic procedure	Per cycle	29.24	—	—	[6]
Indirect cost: productivity loss (absenteeism)		Daily wage	147.24	132.52	161.96	[44]
Discount rate		Annual	0.03	0.00	0.05	N/A
**Cost per cycle and per state**						
	Low-impact LBP^f^	Cycle	441.72	397.55	530.06	See Multimedia Appendix 2
	High-impact LBP	Cycle	588.96	471.17	706.75	See Multimedia Appendix 2
	Treatment weeks 1-4	Cycle	—	—	—	See Multimedia Appendix 2
DTC		—	475.08	335.08	535.08	See Multimedia Appendix 2
TAU^g^		—	377.85	331.40	517.17	See Multimedia Appendix 2
Treatment weeks 4-8		Cycle	16.81	—	—	See Multimedia Appendix 2
Treatment weeks 8-12		Cycle	16.81	—	—	See Multimedia Appendix 2

^a^DTC: digital therapeutic care.

^b^Manually set upper and lower bound values for price level of DTC app cost reimbursement.

^c^GP: general practitioner.

^d^not applicable.

^e^Assuming lower and upper bound values based on a divergent number of physiotherapy sessions: 4 and 12.

^f^LBP: low back pain.

^g^TAU: treatment as usual.

### Sensitivity and Scenario Analyses

We tested all previously mentioned assumptions in a comprehensive deterministic sensitivity analysis (DSA) to validate the robustness of our results. In the DSA, we manually set lower and upper bound values to increase the plausibility of our assumptions. Specifically, we chose divergent remission rates for all dropouts in the range of 40%-60% since it is not clear how many patients discontinue treatment because of sudden pain relief. Regarding the therapy app cost, we set the lower and upper bound values according to alternative price levels for the reimbursement rate of €99.96 and €299.96, respectively. We also varied the number of physiotherapy sessions in the TAU strategy from 4 to 12 to explore the influence of the F2F treatment cost on the overall outcome. For the remainder values, we used confidence intervals as reported in the respective studies or assumed 5% intervals. To address the use of different QoL indices, we tested the SD-6D index values with increased lower and upper bound values of 5%.

We refrained from performing a probabilistic sensitivity analysis (PSA) due to missing data on standard deviations or confidence intervals. Far-reaching assumptions would be necessary that would reduce the value and meaningfulness of a PSA.

Furthermore, we performed several scenario analyses. In scenario A, we simulated different time frames and extended our base case scenario to time horizons of 2 (A.1), 4 (A.2), and 5 (A.3) years. In scenario B, we investigated the impact of three alternative attrition rates (B.1-B.3 as described below) of digital therapeutic care app usage on the overall cost-effectiveness of the intervention compared to TAU. In the base case of our analysis, we used slightly higher attrition rate values in the digital therapeutic care app strategy as adopted from the findings of our reference RCT [18]. However, these numbers were found in a controlled clinical trial environment and do not represent real-world engagement and program dropout rates, which we previously explored in a review of different retrospective studies of digital therapeutic care apps [12].

We previously found divergent attrition rates of digital therapeutic care apps of up to 80% when retrospectively analyzing user databases of real-world app usage frequency [12,46]. Therefore, scenario B extends our base-case analysis by changing the transition probabilities for the digital therapeutic care app strategy in two ways. First, we assumed best-case attrition rates for the digital therapeutic care app strategy to be as low as that in the TAU strategy (B.1). Hence, we decreased attrition rates in the digital therapeutic care strategy to 6.5% after month one and to 4.3% after month two. As alternative worse-case scenarios, we assumed higher attrition rates for the digital therapeutic care strategy with equivalent values after months one and two (B.2: 14% each, B.3: 30% each) to explore the economic consequences when patients are reimbursed for the app but essentially stop using it shortly after.

## Results

In the base-case analysis of our simulation, the digital therapeutic care app strategy cost €121.59 more per patient but also generated additional 0.0221 QALYs compared to the TAU strategy. The incremental cost-effectiveness ratio (ICER) was €5486.05 per QALY. The total expenditures of both the digital therapeutic care and TAU strategies did not significantly differ and amounted to €2039 and €1998 per patient per year, respectively. The indirect cost aggregated to €1442 in the digital therapeutic care strategy and €1550 for TAU. The average QALY values per person and year aggregated to 0.697 in the digital therapeutic care app strategy and to 0.689 for TAU. According to data from a German cost-of-illness study performed by Becker et al [6] in 2010, we addressed 61% of the LBP-related direct cost in our model. By adding the indirect cost components, we addressed 81% of overall health care expenditure resulting from LBP [6]. In the digital therapeutic care strategy, a total of 4143 patients ended up in the “healthy” state, which is 1571 patients more compared with that in the TAU strategy, despite the higher attrition rate. In the TAU strategy, primary care consultations were substantially higher, with a mean number of consultations of 6151 and 5750 per year, respectively. The number of recurring prescriptions for physiotherapy was also 8% higher than the number of app prescriptions. In addition, fewer patients were located in the untreated states “low-impact” and “high-impact” LBP for the digital therapeutic care strategy, leading to a reduced indirect cost. However, the circumstance of the app cost being higher than six sessions of physiotherapy was superior and consequently the reason the digital therapeutic care app strategy was more costly than TAU. The results of the scenario analysis are summarized in Table 3.

**Figure 2:**
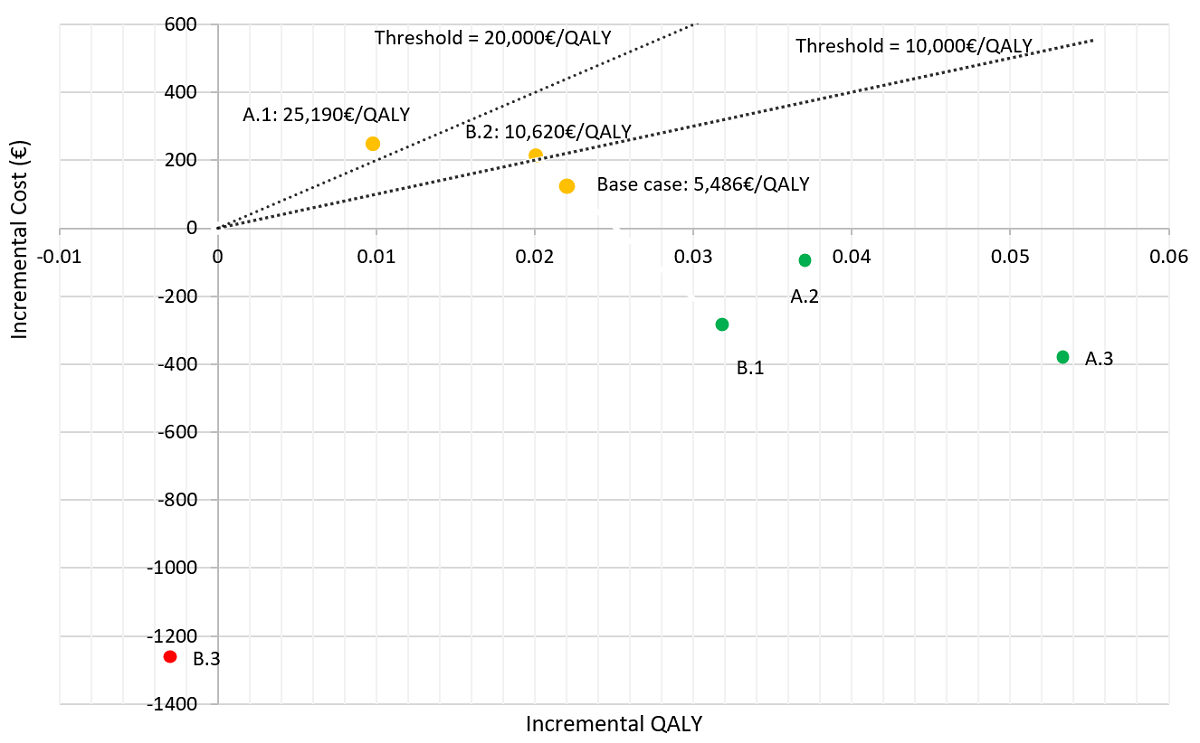
Cost-effectiveness plane, including base case and scenario analyses. The color code indicates when the digital therapeutic care strategy is dominant (green); both scenarios are comparable based on different quality-adjusted life year (QALY) thresholds (orange) or the treatment-as-usual strategy is dominant (red). €1=US $1.12.

The results of our base case and alternative scenarios in the cost-effectiveness plane are visualized in Figure 2. We also included manual threshold values of €10,000 and €20,000 per QALY to provide a better overview regarding the cost-efficiency and thus increase the comparability of the cost per QALY outcome.

**Table 3:** Results of the scenario analyses.

Scenario	Incremental outcome^a^			ICER^b^ result
	Cost outcome^c^	Effect outcome		
A.1: Time horizon 2 years	246.86	0.0098	€25,189/QALY^d^	
A.2: Time horizon 4 years	–99.23	0.0371	DTC^e^ dominant^f^	
A.3: Time horizon 5 years	–381.80	0.0534	DTC dominant	
B.1: Equal attrition rates in both groups (6.5% and 4.3%)^g^	–288.58	0.0319	DTC dominant	
B.2: Higher attrition rates in DTC strategy (14% and 14%)	213.47	0.0201	€10,620/QALY	
B.3: Higher attrition rates in the DTC strategy (30% and 30%)	–1263.62	–0.0029	TAU^h^ dominant	

^a^Incremental outcome referring to the strategy: digital therapeutic care app intervention.

^b^ICER: incremental cost-effectiveness ratio.

^c^Presented in Euro (€1=US $1.12).

^d^QALY: quality-adjusted life year.

^e^DTC: digital therapeutic care.

^f^A dominant strategy: less costly and more generated QALYs.

^g^Monthly attrition rates: 6.5% when transferring from state (3) to (4) and 4.3% in the subsequent cycle when transferring from state (4) to (5).

^h^TAU: treatment as usual.

The different model time horizons were revealed to have a substantial impact on the economic outcome. For the 2-year time horizon (A.1), the cost for one additional gained QALY increased significantly, whereas for the long-term observations (A2 and A3) the app intervention became less costly than TAU and thus the dominant strategy. Moreover, the divergent app attrition rates also showed a strong impact on the outcome. Considering equal attrition rates between app usage and TAU (B.1), digital therapeutic care became the dominant strategy. However, increasing the attrition rate of app users up to 30% (B.3) after each month resulted in TAU becoming the overall dominant strategy.

**Figure 3:**
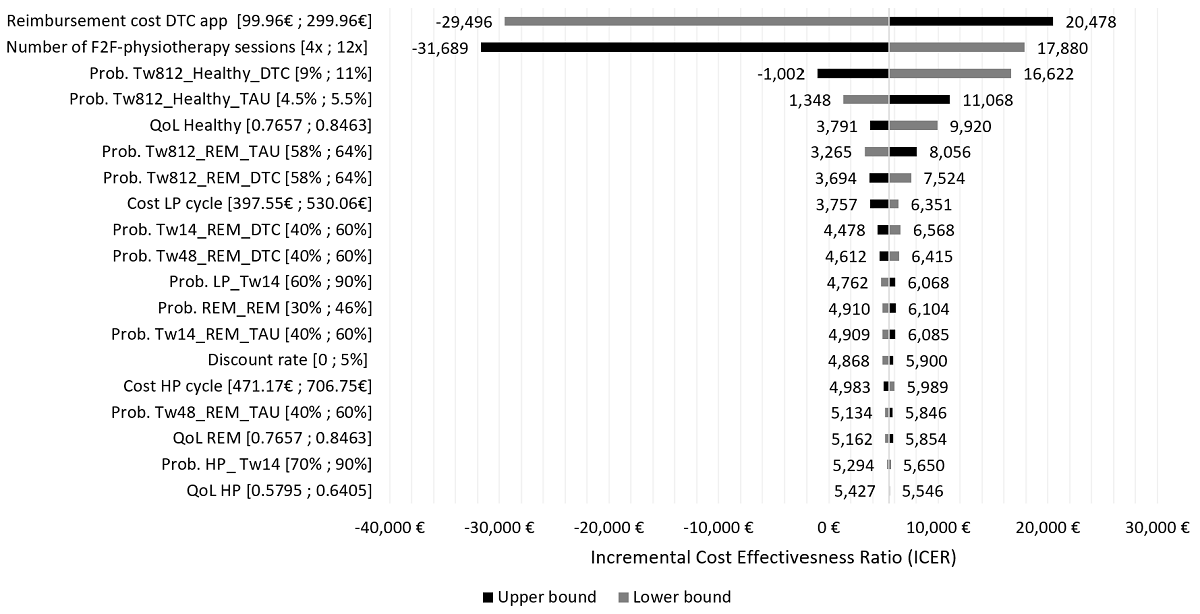
Tornado diagram from the deterministic sensitivity analysis. DTC: digital therapeutic care; F2F: face to face; TAU: treatment as usual; QoL: quality of life; Tw: treatment week; HP: high impact; LP: low impact; REM: remission; Prob: probability of changing states.

The results of our DSA are shown in a tornado diagram centered around the base case result of €5486/QALY in Figure 3. The reimbursement rate of the app and a diverging number of prescribed F2F physiotherapy sessions had the most considerable impact on the results. Increasing the app cost to around €99 per month also increased the cost per generated QALY up to €20,478, while decreasing the app cost to €99 for all 3 months, making digital therapeutic care dominant and significantly less costly than TAU. Similarly, increasing the number of F2F physiotherapy sessions from 6 to 12 within 3 months also made digital therapeutic care dominant over TAU. Except for these two outliers, our simulation results are robust, and parameter uncertainty did not significantly influence our findings.

## Discussion

### Principal Findings

The results of our analysis show that in the base-case analysis, reimbursing a digital therapeutic care app at a rate of €296 for a prescription duration of 3 months results in a cost of €5486 per additional gained QALY compared to TAU. Our decision-analytic model addressed 81% of total direct and indirect costs resulting from LBP and determined an average cost of around €2000 per patient per year in each group. Considering that we excluded inpatient and rehabilitation care, our cost estimate lies in the range of available data from different cost-of-illness studies in Germany with estimates ranging from €1322 and €3580 to €13,745 [5-7]. Our cost outcome value also confirmed our model assumption regarding the amount of absenteeism in the respective health cycles, and subsequently the amount of indirect cost predicted through our model, which averaged at around 70% of the total cost. Although our base-case approach following 8 days of sick leave per cycle is based on scientific findings, we had to make assumptions on when these days of sick leave occur in our model. Hence, we inferred that the majority of days off work occur when patients experience untreated LBP or at the very beginning of the treatment cycle. After treatment begins, medication use enables people to return to work regularly.

The pricing of digital therapeutic care as currently listed in the German DiGa directory is higher than that of six F2F physiotherapy sessions. Concerning commercial apps on the market, therapy app manufacturers offer access to their program for out-of-pocket payers at different price levels, which can vary significantly from around €10 to €99 per month. We have covered this broad range of app pricing within our DSA, in which we defined lower and upper bound values in the range of €99 to €296 for a 3-month treatment. Moreover, therapy apps also have much higher attrition rates, which made it overall unclear if digital therapeutic care is a cost-effective alternative to TAU. Nevertheless, in a digital therapeutic care program, the patients are supported by daily exercise and education material for 12 weeks, in contrast to 45 minutes once per week for 6 weeks of physiotherapy. This also includes personalized decision-support notifications that guide the user, reinforce daily achievements, and thus lead to long-term healthier behavior. It is notable that our explored incremental cost and incremental effect outcomes deviated only by €41 and 0.08 gained QALY per person per year, respectively. Looking at the cost components, the two strategies digital therapeutic care and TAU mainly differ regarding the initial treatment cycle cost, which is the cost of reimbursing a 3-month app prescription versus six F2F physiotherapy sessions. There was no significant between-group difference regarding the VR-6D index values in the QoL data we retrieved from the authors of the RCT [18]. The QoL improvements in the treatment and control group average from 0.671 at baseline to 0.748 after 3 months postintervention in the digital therapy group and from 0.639 to 0.729 in the control group, respectively. Hence, these minor differences ultimately lead to consequential comparable incremental outcomes in the simulation, making a difference only in the long term.

### Determinants for Reimbursement Price Predictions

The scenario and sensitivity analyses revealed the significant drivers and fundamental tradeoffs in our model: the cost of the app and the number of F2F physiotherapy sessions, the impact of digital therapeutic care on behavior change, and overall app attrition rates. We varied both the reimbursement cost of the therapy app and the number of physiotherapy sessions to account for the fact that digital therapeutic care could be equivalent to having up to 12 guided sessions. Both adjustments were found to have the most considerable influence on the ICER of digital therapeutic care. Similarly, the third and fourth bars in the tornado plot of Figure 3 ascertain that personalized health assistance interventions are a decisive factor toward the cost-efficiency outcome of our analysis. We define a decisive factor as an element of the analysis that changes the ICER outcome of our simulation significantly. Since the tornado plot shows that this is the case for these two bars, referring to the transition probability going from state (5) to (7), the effect of decision-support interventions on prolonged behavior change has a substantial impact on the ICER of digital therapeutic care compared to TAU. Conversely, the more people improve their coping behavior with LBP (ie, reach health state 7) in our simulation, the more recurring episodes of LBP can be prevented.

From the perspective of our model, the actual recommended price for reimbursing the app is therefore directly dependent and derivable from two factors: (1) the retention and attrition rates while using the app, and (2) the effect of the implemented decision-support interventions to provoke behavior change and support long-term coping with LBP. If future trials can prove that apps achieve lower attrition rates in real-world usage as they currently do in a controlled clinical trial environment, our analysis confirms that digital therapeutic care becomes dominant over in-person physiotherapy. Moreover, if apps can support behavior change and the patient’s self-management of LBP, our analysis confirms that the cost per generated QALY decreases for the digital therapeutic care strategy. Both factors significantly impact the formation and reevaluation of the reimbursement price and thus determine the amount of profit contribution for the app developers.

It is inevitable that the level of benefit and the patient’s perception of the actual value of digital therapeutic care ultimately determine future reimbursements rates. Hence, more clinical effectiveness trials, including patient-reported outcome measures, are needed to generate more insights on these critical factors to further increase our model’s usefulness and make actual price predictions for the statutory health insurance.

### Need for Further Cost-Effective Analyses Considering Different Patient Cohorts and Scenarios

In our model, we focused on a specific use case that implies the prescription of the therapy app to patients with subacute or chronic LBP as an alternative modality to in-person physiotherapy. However, there are other application areas that amplify the advantages of using an app and could potentially increase the cost-effectiveness of digital therapeutic care significantly. A cluster randomized trial has found that stratified care or immediate access to the app without prolonged waiting times on F2F physiotherapy is highly effective in preventing the worsening of LPB to a chronic condition. Thereby, early reduction of overall persistent pain levels could have a tremendous positive impact on the economic burden of LBP [16].

Moreover, we based our analysis on an RCT that has elaborated the efficiency of a multidisciplinary therapy app, including exercises, education material, and push notifications for people with LBP [18]. Considering the multidisciplinary capabilities of digital therapeutic care and the fact that multimodal offline rehabilitation programs are much more expensive in Germany, therapy apps could be a cost-effective alternative to these offline resource-extensive programs. It remains unclear if future effectiveness trials could show that therapy apps are as efficient as a multimodal rehabilitation program or if digital therapeutic care also provides synergistic effects as an add-on supporting modality. Subsequently, future studies should further explore how other therapeutic apps with different in-app features, dashboards, or even backends should be treated, and if cost-effectiveness analyses have to be conducted individually for each app based on the respective clinical effectiveness data.

Finally, our study relied on single study–based estimates with a small-sized and narrow cohort. In our simulation, we assumed a middle-aged cohort with above-average education and a medium BMI, thus possibly constraining the transferability of our results to a broader population. Coping with LBP by self-managing the digital therapeutic care program may be more challenging for other populations. For example, people with a higher BMI might experience more insecurities and fear-avoidance beliefs and drop out earlier, or less educated people might find it challenging to understand and adopt the necessity of behavior change [47]. Hence, further economic evaluations, including different patient characteristics, scenarios, and control groups, are required to make a profound conclusion on the cost-efficiency for including digital therapeutic care apps into the statutory health insurance.

### Transferability of Results to Other Countries

The lack of transferability also applies for implications toward the cost-efficiency of alternative health care systems in other countries. Our model’s input data are specifically tailored toward the addition of digital therapeutic apps into the German statutory health insurance reimbursement catalog by drawing on insights from various German cost-of-illness studies. However, efficiency studies from other therapy apps for people with LBP have proven similar positive health effects on QoL and pain intensity, and might thus imply the same impact on the economic outcome [12]. The fact that our results imply an ICER of €5486 per QALY is highly promising for other countries, such as the UK National Institute for Health and Care Excellence in England with a cost-effectiveness threshold in the range of £20,000 to £30,000 (US $26,764 to US $40,146) per QALY [48].

### Comparison With Prior Work

Prior model-based economic evaluations concerning the long-term cost-efficiency of various treatment interventions and management of LBP were found to be sparse and with a poor standard of modeling [49,50]. A recent systematic review performed by Hall et al [49] identified a total of five studies that encompass a health economic decision model (ie, based on a decision tree or state transition for any treatment modality for LBP) [49]. The authors concluded a predominantly poor quality of modeling techniques, especially regarding the applied health states concerning a suboptimal representation of LBP health conditions and treatment pathways or inadequate time horizons and model cycle lengths [49]. Remarkably, concerning the three Markov model–based studies in their review, all constructed models included a total of three or four health states each to represent the respective treatment approach, which might entail oversimplification biases. Among the studies in their review, the authors favored a state-transition model in which the initial health states served as a temporal classification of LBP (eg, “acute,” “subacute,” or “chronic” LBP, and “healthy”) [51]. However, the heterogeneity of pain severity and functional disability cannot sufficiently be reflected in a chronological arrangement of health states, while recurrent LBP episodes could result in a false state classification. Therefore, a modeling approach considering the severity of symptoms (eg, focusing on the level of pain) is recommended as the current best course of action [52].

In advance of our economic evaluation, we performed a snowball sampling search method to complement the view from the review of Hall et al [49] with any more recently published studies, allowing us to draw further insights on best-practice modeling techniques in this field. We searched the reference lists and used Google Scholar’s “cited by” function to find additional model-based studies concerning any treatment interventions for LBP. In total, we found another four publications, including three distinct Markov models [52-55]. First, Hall et al [52] performed a cost-utility analysis exploring the STarT Back stratified care model compared to usual care. Based on a six-health-state transition model, the authors concluded that stratified care is cost-effective for managing LBP over a 10-year horizon [52]. Second, Hermann et al [53] constructed a four-health-state transition model and investigated the cost-efficiency of 17 nonpharmacologic therapies compared to usual care over a 1-year time horizon. The authors updated their model in a subsequent publication by adding five additional trials with further alternative treatment modalities into their analysis [53,54]. Lastly, to complete the list of related work regarding available Markov model–based economic analyses of managing LBP, Olafsson et al [55] constructed a hybrid decision tree state-transition model to establish a lifetime treatment pathway model based on Swedish national registry data to extrapolate a mean lifetime total cost of €47,452 per LBP patient. We analyzed all additional studies more profoundly according to the individual model approaches, model conceptualization, and underlying techniques, which are summarized in Multimedia Appendix 3. Economic evaluations alongside clinical randomized controlled or observational trials are more prevalent and have been summarized for various LBP treatment modalities in numerous systematic reviews published in the previous 3 years alone [56-59].

### Limitations

Our model and analyses have several limitations and constraints. First, we did not include all cost components and treatment dimensions related to LBP. By addressing only 81% of LBP occurred costs and excluding inpatient and rehabilitation care, we may have caused over- or underestimation of costs and neglected the coherences and impact of digital therapeutic care on resource utilization of alternative treatment modalities. Although we specifically included the interventions recommended by German treatment guidelines, we excluded other minor yet prevalent applied interventions in outpatient care, such as injections therapy, because we would lack relevant data to integrate this alternative pathway into our model [35]. The high numbers of different options and treatment considerations make it challenging to develop a model that considers a broader patient cohort than we did in our simulation [50].

Furthermore, we did not perform a PSA because of the lack of information. For most of our input parameters, essential data such as standard deviations or confidence intervals were not available so that using a recommended beta or gamma distribution in the PSA was not feasible. Another limitation is the use of two different metrics, the SF-6D and VR-6D, as part of the QoL measurement of effectiveness. Although we do not expect our results to differ significantly because these two metrics provide comparable indices and the health states with the SF-6D utility values are equal for both strategies, this methodological choice is a limitation of our study. Finally, we emphasize that the QoL data of the treatment health states are taken from one RCT and are not derived from synthesis-based estimates such as a meta-analysis of QoL effectiveness studies, since more data are not yet currently available in the scientific literature. Although we tested all relevant uncertain parameters within our sensitivity analysis, more research is required on the cause and consequences of fluctuating LBP intensity as well as the reasons behind early and spontaneous treatment discontinuations.

### Conclusion

We developed a best-practice model for evaluating the cost-effectiveness of digital therapeutic care compared to TAU for people with LBP, and provided the first long-term economic evidence for reimbursing an app by the statutory health insurance in Germany. The current reimbursement cost set at €296.99 for a 3-month app prescription can be considered cost-effective compared to TAU with an ICER of €5486 per generated QALY. Future value-based price targets should focus on additional outcome parameters besides the effect on the QoL or reduction in pain intensity. Including the app’s attrition rate and the effect on the patient’s coping ability and behavior change induced by the app’s personalized assistance interventions will essentially influence the setting of a holistic value-based reimbursement price.

## Appendix

Multimedia Appendix 1 Items to include when reporting economic evaluations of health interventions (Consolidated Health Economic Evaluation Reporting Standards).

Multimedia Appendix 2 Markov model input parameters.

Multimedia Appendix 3 Overview of related work.

## Abbreviations

CHEERS: Consolidated Health Economic Evaluation Reporting Standards
DiGa: Digital Health Applications directory
DSA: deterministic sensitivity analysis
F2F: face to face
GP: general practitioner
ICER: incremental cost effectiveness ratio
LBP: low back pain
PSA: probabilistic sensitivity analysis
QALY: quality-adjusted life year
QoL: quality of life
RCT: randomized controlled trial
SF-6D: Short Form 6-Item Health Survey
TAU: treatment as usual
VR-6D: Veterans RAND 12-Item Health Survey
VR-12D: Veterans RAND 12-Item Health Survey
