# Supplementary material for: Digital Therapeutic Care Apps With Decision-Support Interventions for People With Low Back Pain in Germany: Cost-Effectiveness Analysis
Source: JMIR Mhealth Uhealth. 2022 Feb 7;10(2):e35042. doi: 10.2196/35042 (PMC8861873; doi:10.2196/35042)
Supplement: Multimedia Appendix 2 [file mhealth_v10i2e35042_app2.docx]

**Multimedia Appendix 2: Markov Model Input Parameter.**

Initial health states starting population.

The initial starting population data is based on the distribution of the Von Korff Graded Chronic Back Pain Status (GCPS) and data is extracted from the following two studies: Schmidt et al. [3] and Wenig et al [5]. We combined GPCS categories 1 with 2 as well as 3 with 4 (also referred to as “disabling back pain” categories) by adding up the percentage of the retrospective frequency distribution from both studies.

We thus receive the following starting population (10,000) [5].

GCPS Grade 0 = 35.6% in state (6) Remission --> 3,560

GCPS Grade 1 (45.2%) + GCPS Grade 2 (8.0%) = 53.2% in state (1) Low impact --> 5,320

GCPS Grade 3 (6.6%) + GCPS Grade 4 (4.6%) = 11.2% in state (2) High impact --> 1,120

Transition probabilities

| **Parameter** | **Base Case** | | | |
| --- | --- | --- | --- | --- |
|  | **DTC App** | | **TAU** | |
| Absolute transition probabilities before final calculation | |  |  |  |
| Low impact -> Low impact | 80% | | | |
| Low impact -> High impact | 5% | | | |
| Low impact -> Treatment w1-4 | 75% | | | |
| Low impact -> Remission | 15% | | | |
| High impact -> Low impact | 21% | | | |
| High impact -> High impact | 79% | | | |
| High impact -> Treatment w1-4 | 80% | | | |
| High impact -> Remission | 0 | | | |
| Treatment w1-4 -> Low impact | 82.2% | | | |
| Treatment w1-4 -> High impact | 17.8% | | | |
| Treatment w1-4 -> Treatment w4-8 | 87.5% | | | 93.5% |
| Treatment w1-4 -> Remission | 50% | | | |
| Treatment w4-8 -> Low impact | 82.2% | | | |
| Treatment w4-8 -> High impact | 17.8% | | | |
| Treatment w4-8 -> Treatment w8-12 | 87.5% | | | 95.7% |
| Treatment w4-8 -> Remission | 50% | | | |
| Treatment w8-12 -> Low impact | 82.2% | | | |
| Treatment w8-12 -> High impact | 17.8% | | | |
| Treatment w8-12-> Remission | 61.4% | | | |
| Treatment w8-12 -> Healthy | 10% | | | 5% |
| Remission -> Remission | 38.6% | | | |
| Remission -> Low impact | 82.2% | | | |
| Remission -> High impact | 17.8% | | | |

Calculation of the quality of life (QoL) input parameters

After retrieving the data, which are the results from the VR-12 survey, from the DTC app RCT [5], we calculated the VR-6D according to Selim et al approach [45].

We calculated the following VR-6D parameter:

| Strategy | T_0: at baseline | T_1: at 6 weeks | T_2: at weeks |
| --- | --- | --- | --- |
| DTC | 0.671 | 0.707 | 0.747 |
| TAU | 0.639 | 0.708 | 0.729 |

In order to enable a same level QoL starting value for health state (3), we additionally performed a simple regression calculation and applied the rule of three in order to equalize the values at baseline.

After adjusting the VR-6D, we retrieved the following QoL income parameter for our model:

| Strategy | T_0: at baseline | T_1: at 6 weeks | T_2: at weeks |
| --- | --- | --- | --- |
| DTC | 0.655 | 0.698 | 0.748 |
| TAU | 0.655 | 0.717 | 0.729 |

Cost per cycle calculation

In the following, we describe in detail the single cost components as listed in Table 2 in the manuscript and how we calculated the costs per cycle in our model:

| **Cost per Cycle and per state** | | | |  |
| --- | --- | --- | --- | --- |
| Low impact LBP | Cycle | 441.72 | = 3 days (average productivity loss per cycle) x 147.24€ (daily wage) | |
| High impact LBP | Cycle | 588.96 | = 4 days (average productivity loss per cycle) x 147.24€ (daily wage) | |
| Treatment week 1-4 | Cycle |  |  | |
| **DTC** |  | 475.08 | = 239.96€ (current reimbursement rate in Germany) + 20.47€ (consultation GP) + 21.36€ (consultation specialist) + 16.81€ (medication) + 29.24€ (diagnostic procedures) + 147.24€ (average 1 day sick from work) | |
| **TAU** |  | 377.85 | = 20.47€ (consultation GP) + 21.36€ (consultation specialist) + 16.81€ (medication) + 29.24€ (diagnostic procedures) + 147.24€ (average 1 day sick from work) + 149.33€ (cost of six in-person physiotherapy sessions)  // Cost of six physiotherapy session: (6x21.11€) + 10% + 10€ | |
| Treatment  weeks 4-8 and 9-12 | Cycle |  |  | |
| **DTC** |  | 16.81 | Mean medication cost per month. | |
| **TAU** |  | 16.81 | Mean medication cost per month. | |
