# Supplementary material for: Digital Therapeutic Care Apps With Decision-Support Interventions for People With Low Back Pain in Germany: Cost-Effectiveness Analysis
Source: JMIR Mhealth Uhealth. 2022 Feb 7;10(2):e35042. doi: 10.2196/35042 (PMC8861873; doi:10.2196/35042)
Supplement: Multimedia Appendix 3 [file mhealth_v10i2e35042_app3.docx]

**Multimedia Appendix 3:** Model-based economic evaluations for the management of LBP.

| **Number and names of**  **health states** | (7): Low Risk, Medium Risk, High Risk, Good Function, Moderate Function, Poor Function, Dead | (4): High-impact chronic pain, no pain, higher (moderate) impact pain, lower (low-impact) pain | In total 10 health states following the decision tree structure:  (4): Symptoms resolved, sick leave, conservative care, and inpatient care.  (6): In Markov post-surgical care |
| --- | --- | --- | --- |
| **Horizon** | 10 years | 1 year | Lifetime |
| **Cycle length** | 2 months | 6 weeks | 3 months |
| **Model type** | Markov state-transition model | Markov state-transition model | Markov state-transition model and decision tree |
| **Control group** | Usual care | Usual care | - |
| **Interventions** | Stratified care model (STarT Back) | 17 + 5 Non-pharmacologic interventions for chronic low back pain | Lifetime treatment pathway model |
| **LBP population** | 55 years old, 57% female LBP patients | Chronic LBP patients | 154,209 patients, mean age 50, with any kind of LBP |
| **Perspective** | UK National Health Service & Societal perspective | Societal and payer perspective | Societal perspective |
| **Country** | UK | US cost  (Efficacy trials also from Canada or UK) | Sweden |
| **Authors/**  **Year** | Hall et al.  2020 | Herman et al.  2019 +  Herman et al.  2020 | Olafsson et al.  2017 |
